# Fasting-Mimicking Diet Reduces Trimethylamine N-Oxide Levels and Improves Serum Biochemical Parameters in Healthy Volunteers

**DOI:** 10.3390/nu14051093

**Published:** 2022-03-05

**Authors:** Melita Videja, Eduards Sevostjanovs, Sabine Upmale-Engela, Edgars Liepinsh, Ilze Konrade, Maija Dambrova

**Affiliations:** 1 Latvian Institute of Organic Synthesis, LV-1006 Riga, Latvia; eduards@osi.lv (E.S.); upsabine@farm.osi.lv (S.U.-E.); ledgars@farm.osi.lv (E.L.); maija.dambrova@farm.osi.lv (M.D.); 2 Faculty of Pharmacy, Riga Stradiņš University, LV-1007 Riga, Latvia; 3 Department of Endocrinology, Riga East University Hospital, LV-1038 Riga, Latvia; ilze.konrade@rsu.lv; 4 Department of Internal Diseases, Riga Stradiņš University, LV-1007 Riga, Latvia

**Keywords:** trimethylamine N-oxide, fasting-mimicking diet, weight loss, insulin sensitivity, cardiometabolic risk

## Abstract

Elevated plasma levels of trimethylamine N-oxide (TMAO) have been proposed as a diet-derived biomarker of cardiometabolic disease risk. Caloric restriction is the most common dietary intervention used to improve cardiometabolic health; however, novel trends suggest a fasting-mimicking diet (FMD) as a more feasible alternative. FMD is a variation of intermittent fasting, based on caloric restriction and limitation of protein sources of animal origin, applied in daily cycles during a 5-day period. As TMAO is intensively produced by gut microbiota after the consumption of animal-derived products, we aim to investigate whether a 5-day FMD affects plasma TMAO levels and markers of metabolic health. To investigate whether an increase in vegetable intake possesses similar effects on TMAO levels and metabolic parameters, healthy volunteers (*n* = 24) were subjected to a 5-day FMD and 19 volunteers served as a reference group (VEG). This group of volunteers consumed an additional four servings of vegetables per day, but otherwise stayed on their usual diet. FMD resulted in a twofold decrease in plasma TMAO levels, which was not evident in the volunteers from the VEG group. Moreover, FMD led to a weight loss of 2.8 ± 0.2 kg and a subsequent reduction in BMI compared to baseline. The FMD group exhibited a significant elevation in plasma ketone bodies (14-fold compared to baseline) and a decrease in IGF-1 levels by 37 ± 8 ng/mL. Since fasting glucose and C-peptide levels decreased, all volunteers in the FMD group showed improved insulin sensitivity and a decreased HOMA-IR index. In contrast, in the VEG group, only a slight reduction in plasma levels of fasting glucose and triglycerides was noted. In conclusion, we show that FMD is a viable strategy to reduce plasma levels of TMAO by limiting caloric intake and animal-derived protein consumption. The reduction in the level of TMAO could be an additional benefit of FMD, leading to a reduced risk of cardiometabolic diseases.

## 1. Introduction

Despite the well-known relationship between unhealthy dietary patterns and an increased risk of cardiovascular and metabolic diseases, research continuously tries to identify novel diet-derived culprits that are responsible for the molecular mechanisms that cause the detrimental health effects. One study that attempted to link dietary choices and cardiometabolic health identified that the gut microbiota metabolite trimethylamine N-oxide (TMAO) is associated with a higher incidence of adverse cardiovascular outcomes [1]. In recent years, TMAO concentrations have been extensively studied in various patient populations. Since its initial discovery, strong associations have been reported between systemic TMAO levels and coronary artery atherosclerosis [2,3,4,5], which is known to be the leading cause of CVDs. Other possible mechanisms linking TMAO to the pathogenesis of CVDs include platelet activation, increased probability of thrombosis [6], aggravation of vascular inflammation [7,8] and prolongation of the hypertensive effect of angiotensin II [9], as indicated by preclinical research. Clinical studies, in turn, have added to this knowledge by identifying a TMAO plasma concentration of 6.18 μmol/L as a major adverse cardiovascular event risk threshold [10]. An increase in TMAO plasma levels also correlates with heart failure severity according to the New York Heart Association (NYHA) classification (NYHA II-3.5 ± 0.9; NYHA III-6.0 ± 0.8; NYHA IV-8.1 ± 1.0 µmol/L, respectively) [11] and correlates with the advancement of T2D [12]. Subsequently, T2D patients with elevated plasma levels of TMAO are also more susceptible to major adverse cardiovascular events, such as myocardial infarction, hospitalization for heart failure and unstable angina [13]. In addition, a dose–response meta-analysis revealed that the relative risk for all-cause mortality increased by 7.6% per 10 μmol/L increase in TMAO levels [14].

The initial step of TMAO formation occurs in the intestines, where a variety of gut microbial enzymes metabolize dietary precursors, such as L-carnitine, betaine and choline, to form trimethylamine (TMA). These precursors of TMA are highly abundant in protein sources of animal origin, such as red meat, liver, high-fat dairy products and eggs as well as some legumes [2,10]. Furthermore, TMA is oxidized to TMAO in the liver by the enzyme group flavin-containing monooxygenases (FMOs) [2,15]. Moreover, fish and other seafood contain high concentrations of TMAO; therefore, the consumption of marine products can also increase plasma levels of TMAO [16]. To date, it has been shown that a diet rich in saturated fat modifies the gut microbiota and leads to increased TMAO levels in rodents [17] and humans [18]. Moreover, the adherence to a Western-style diet also results in impaired cardiac function, which can be prevented if TMA formation is inhibited pharmacologically [8]. Thus far, such pharmacological means as antibiotics, metformin, meldonium and structural analogues of choline have been considered as possible TMAO-lowering strategies [19,20]. However, the pharmacological inhibition of TMAO production cannot prevent disturbances in the lipid profile and obesity [8], indicating that, in addition to pharmacological intervention, further lifestyle changes would still be necessary. Therefore, studying dietary approaches targeting the level of TMAO together with other metabolic parameters would be of great significance.

To date, caloric restriction has been the most well-known and widely applied dietary strategy; it has been used to achieve a healthy weight, improve metabolic health and promote longevity in humans [21,22]. However, problems with long-term compliance with caloric restriction have been identified in clinical studies [23,24,25]. Recently, intermittent fasting has gained scientific interest as a novel dietary regimen with the aim of improving metabolic health [26]. The main strategies of intermittent fasting rely either on restriction of food intake for periods ranging from 16 to 48 h (time-restricted fasting, alternate-day fasting, or 5:2 days cycle) or the reduction in total caloric intake and abstaining from specific macronutrients (fasting-mimicking diet (FMD)) [27]. FMD is a hypocaloric, vegetable-based diet with strictly limited animal protein intake, and it is applied in cycles of 5 subsequent days a month [28]. Recent studies have shown the beneficial effects of FMD on cardiovascular disease (CVD) risk markers, such as BMI, total and trunk body fat, systolic and diastolic blood pressure and insulin resistance [29]. This type of periodic energy restriction imitates the metabolic patterns of prolonged fasting; however, it is easier to comply with and safer than the complete cutback of calories [28].

Although there is some evidence that TMAO levels could be targeted by some types of caloric restriction [30,31,32], reduced protein intake [33] or diets supplemented with sources of dietary fibers and unsaturated fatty acids [34,35], it has not been thoroughly investigated. As FMD has shown potential in reducing CVD risks, our objective is to investigate whether this short-term reduction in caloric intake by decreasing the consumption of animal-derived proteins, as in the case of FMD, could also serve as an effective dietary strategy to reduce plasma TMAO levels in healthy omnivorous volunteers. As FMD is based on a high vegetable intake, we compared the clinical biochemistry measurements of the volunteers undergoing the cycle of FMD to those who incorporated additional amounts of vegetables in their usual diet. This was to specify that the effects of FMD are attributed to intermittent energy restriction and the reduction in protein, not the high abundance of vegetables in the diet. As a measure of volunteer compliance, we assessed the levels of plasma ketone bodies and insulin-like growth factor-1 (IGF-1), parameters that are usually affected by prolonged fasting and reduced protein intake.

## 2. Materials and Methods

### 2.1. Volunteers

A total of 44 omnivorous volunteers were subjected to an interventional study approved by the local Ethics Committee of Riga Stradiņš University, Latvia (No. 6-2/10/51). Routine biochemistry tests and blood counts were performed to assess the general health of all volunteers prior to joining the study. The exclusion criteria were as follows: BMI < 18.5 kg/m^2^; abnormal levels in any of the blood biochemistry measurements that indicate severe health problems; and taking antibiotics, probiotics or dietary supplements containing TMAO precursors within 2 months before the start of dietary interventions. All volunteers were informed about the aim and nature of this study. The recruitment of the volunteers and study procedures were carried out between December 2019 and June 2021.

### 2.2. Study Design

**Figure 1:**
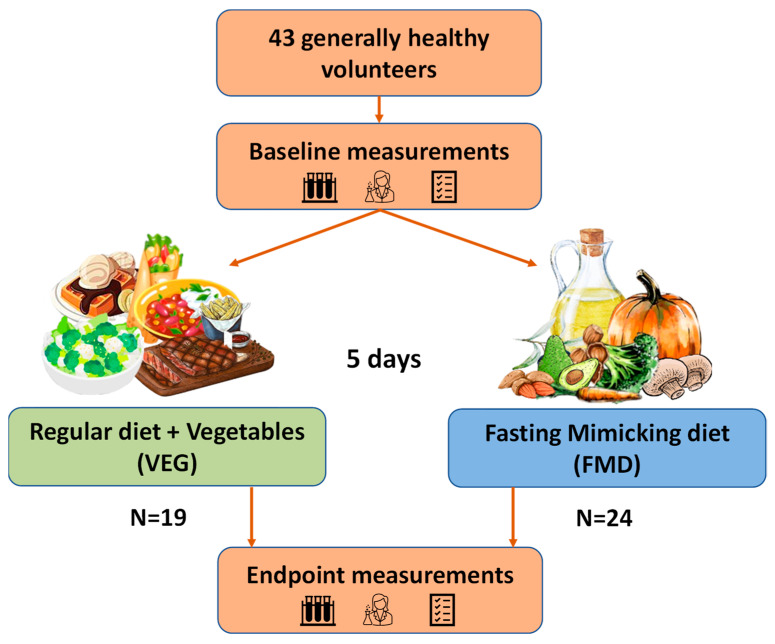
Schematic representation of the study design.

The schematic design of the study is presented in Figure 1. Baseline anthropometric measurements and biochemical tests were performed in a fasted state before the planned dietary intervention. All participants were instructed to fast ≥ 10 h prior the blood sampling; drinking pure water was allowed during the fasting time. As fish consumption could interfere with the measurement of the TMAO level, volunteers were asked to abstain from sea food consumption for two days prior to sampling. Participants were asked to maintain their usual levels of physical activity throughout the intervention. The research was carried out as a parallel arm study, and the volunteers were assigned to either the reference group (VEG) or fasting-mimicking diet (FMD) group for 5 days. The baseline characteristics of the participants are presented in Table 1. Fasting plasma glucose was chosen as the main parameter for the randomization of the volunteers.

FMD as a dietary regimen was based on the plan developed by the team of Prof. Valter D. Longo [28]. Briefly, participants in the FMD group were subjected to a 5-day hypocaloric diet that provides 34–54% of regular caloric intake (approximately 1100 kcal on the first day and approximately 800 kcal on the four subsequent days). The volunteers in the FMD group were asked to consume primarily complex carbohydrates and unsaturated fat, but to limit protein intake (the caloric intake of these macronutrients was distributed as follows: 40–45%; 45–50%; 10–15%, respectively). The meals in the FMD group mainly consisted of vegetables, seeds, nuts and vegetable oils. Legumes were allowed only on the first day as they are considered a protein source.

Volunteers in the VEG group were expected to continue their usual dietary regimen, with the exception that they were asked to incorporate 4 servings (each approximately 100–125 g) of vegetables into their diet per day. The sizes of the meals, the caloric intake and the macronutrient content of the diet were otherwise unrestricted.

The volunteers subjected to this interventional study were under careful supervision throughout the study. Detailed information leaflets were prepared and distributed to volunteers, containing all the important information about the dietary intervention to which they were assigned and the list of allowed products together with their nutritional value. Volunteers usually ate two identical meals together (breakfast and lunch), For the evaluation of dinner, a special WhatsApp Messenger group was created, where volunteers shared photos of their meals prepared at home, which was also used as a measure of volunteer compliance.

After 5 days of dietary intervention, volunteers were weighed and blood samples in the fasted state (fasting at least 10 h prior the blood sampling) were taken. One volunteer from the FMD group withdrew from the study due to difficulties adhering to the dietary regimen. Samples previously taken from this volunteer were excluded from further analysis.

### 2.3. Determination of Biochemical Measurements

Blood sampling was carried out in the fasted state immediately before the start of the dietary intervention and the morning after the 5-day dietary intervention. The samples obtained were stored on ice and delivered to the Limited Liability Company “E. GULBJA LABORATORIJA” (accredited by the Latvian National Accreditation Bureau, accreditation No. M-365) within two hours. The samples were subjected to clinical chemistry analyses. β-Hydroxybutyrate (plasma ketone bodies) was measured using a commercially available enzymatic kit (Biosystems S. A, Barcelona, Spain; Lot 39099) according to the manufacturer’s instructions. Briefly, the obtained plasma was 5-fold diluted. The standard curve was generated from 3-Sodium hydroxybutyrate (Alfa Aeser, Ward Hill, MA, USA) and assayed in duplicate. All samples were assayed in duplicate. The enzyme assay is based on oxidation of β-Hydroxybutyrate in the presence of NAD+, to form acetoacetate and NADH. The NADH produced is further involved in an indicator reaction that results in the formation of formazan that can be detected spectrophotometrically [36].

### 2.4. Measurement of TMAO Levels by UPLC/MS/MS

The concentration of TMAO in plasma samples was determined by ultraperformance liquid chromatography tandem mass spectrometry (UPLC/MS/MS) using the positive ion electrospray mode described previously [37,38]. In brief, the obtained blood samples were centrifuged at 3000× *g* for 5 min at 4 °C to separate plasma. Plasma samples were collected and stored at −80 °C, until further analysis. The samples were prepared for further analyses by deproteinization with an acetonitrile–methanol mixture (3:1, *v*/*v*), followed by vortexing and centrifugation at 13,000× *g* for 10 min. The supernatant was transferred to UPLC vials and used for UPLC/MS/MS analysis. MassLynx 4.1. software with the QuanLynx 4.1. module (Waters, Milford, PA, USA) was used for data acquisition and processing. A sample of an original data file of TMAO detection using UPLC/MS/MS analysis is available in Supplementary Table S1.

### 2.5. Data Analysis

The calculation of insulin sensitivity and insulin resistance indices was performed using homeostatic model assessment and HOMA2 Calculator (version 2.2.3, available online, developed by Diabetes Trial Unit, University of Oxford, Oxford, U.K.) [39].

Statistical analysis of the data was performed using GraphPad Prism computer software (GraphPad, Inc., San Diego, CA, USA). The results are reported as the mean ± SEM. Statistical significance between two groups was evaluated using paired Student’s *t*-test or Wilcoxon matched-pairs test, depending on the data distribution, which was determined using Shapiro–Wilk test. Differences were considered significant when the two-sided *p* value was below 0.05.

## 3. Results

All recruited volunteers were generally healthy, as baseline biochemistry measurements did not indicate any severe health-related conditions of any of the organ systems. Anthropometric measurements, on the other hand, suggested that the volunteers were slightly overweight with a mean BMI of 27.2 ± 0.7 units. The mean TMAO concentration in plasma was 5.08 ± 0.74 μmol/L at baseline.

**Figure 2:**
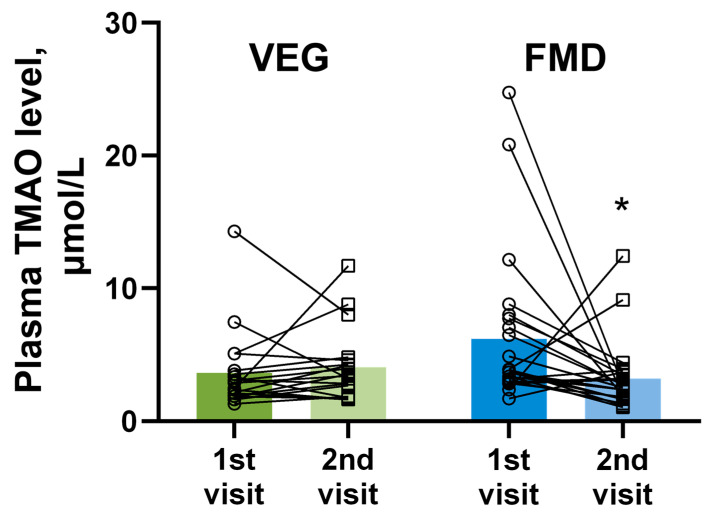
The impact of the 5-day cycle of regular diet supplemented with 4 servings of vegetables (VEG) and fasting-mimicking diet (FMD) on the plasma level of trimethylamine N-oxide (TMAO) in healthy volunteers. The results are presented as the mean and independent values of 19 volunteers in the VEG group and 24 volunteers in the FMD group. * Indicates a significant difference from the respective group at the 1st visit (Wilcoxon matched-pairs test), *p* < 0.05.

The measurement of plasma TMAO levels (Figure 2) revealed that 5 days of the regular diet supplemented with four servings of vegetables per day (VEG) did not result in significant changes in plasma TMAO levels, with a mean increase of 0.43 ± 0.70 μmol/L. In 8 out of 19 volunteers, we observed a reduction in plasma TMAO levels after the dietary intervention; however, 11 volunteers experienced an increase in plasma TMAO levels. In contrast, 75% (18 out of 24) of the volunteers who followed the FMD experienced a notable reduction in plasma TMAO levels. Despite the fact that the interindividual variability of the baseline TMAO levels was high, we observed a strong correlation between the plasma TMAO levels at baseline and the decrease in plasma TMAO levels for those who underwent 5 days of FMD. Moreover, the average plasma level of TMAO in the FMD group at the second visit was 3.01 ± 1.43 μmol/L lower than that at the first visit.

**Figure 3:**
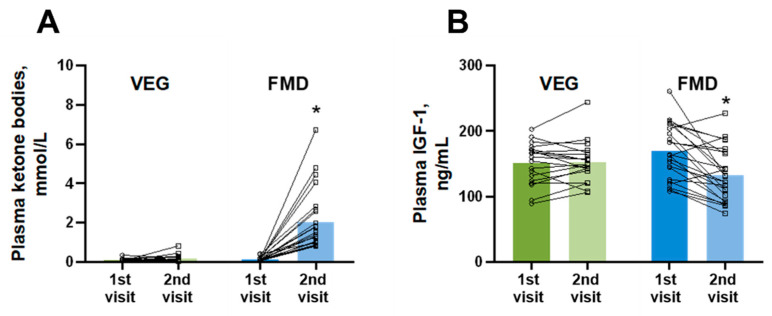
Changes in the levels of plasma ketone bodies (**A**) and insulin-like growth factor-1 (IGF-1) (**B**) induced by 5 days of the regular diet supplemented with additional vegetables (VEG) and fasting-mimicking diet (FMD). The results are presented as the mean and independent values of 19 volunteers in the VEG group and 24 volunteers in the FMD group. * Indicates a significant difference from the respective group at the 1st visit (Wilcoxon matched-pairs test), *p* < 0.05.

As FMD as a dietary regimen is based on imitating the molecular effects of prolonged fasting, we next evaluated the effects of both diets on plasma ketone body concentrations. As shown in Figure 3A, 5 days of the VEG diet resulted in only a slight increase in plasma ketone body levels from 0.11 ± 0.02 mmol/L to 0.16 ± 0.04 mmol/L. In contrast, the FMD group exhibited a significantly higher increase in plasma ketone body levels by 1.87 ± 0.32 mmol/L (14-fold elevation compared to baseline measurement). We also observed a significant reduction in plasma insulin-like growth factor-1 (IGF-1) concentrations (Figure 3B) in the FMD group by 37 ± 8 ng/mL, which was not present in the VEG diet group.

**Figure 4:**
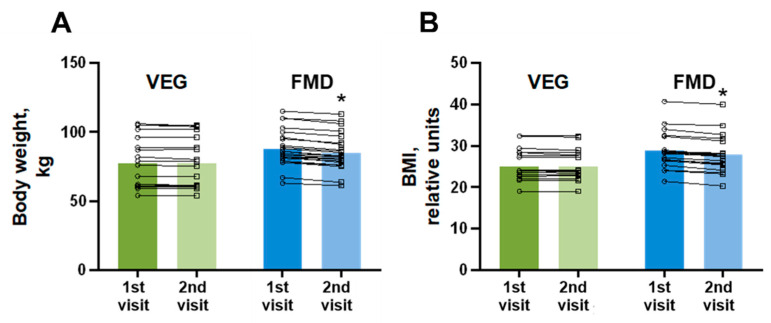
Effects of a 5-day regular diet with additional intake of vegetables (VEG) and fasting-mimicking diet (FMD) on weight (**A**) and BMI (**B**) in healthy volunteers. The results are presented as the mean and independent values of 19 volunteers in the VEG group and 24 volunteers in the FMD group. * Indicates a significant difference from the respective group at the 1st visit (Wilcoxon matched-pairs test), *p* < 0.05.

To investigate the contribution of applied dietary strategies to weight loss, the volunteers were weighed before the study and after 5 days of the applicable diet. At baseline, volunteers in the VEG group weighed 78 ± 4 kg. The baseline weight of the volunteers in the FMD group was slightly higher (88 ± 3 kg). Only five volunteers subjected to the VEG diet experienced a slight weight reduction of an average of 0.28 ± 0.15 kg of body weight (Figure 4A). However, each of the volunteers who followed FMD experienced significant weight loss. The average weight loss in the FMD group after 5 days of the dietary intervention was 2.8 ± 0.2 kg of body weight. These changes in body weight resulted in a more pronounced reduction in the body mass index (Figure 4B) in the FMD group (0.90 ± 0.06 units in the FMD group compared to 0.09 ± 0.05 units in the VEG group).

**Figure 5:**
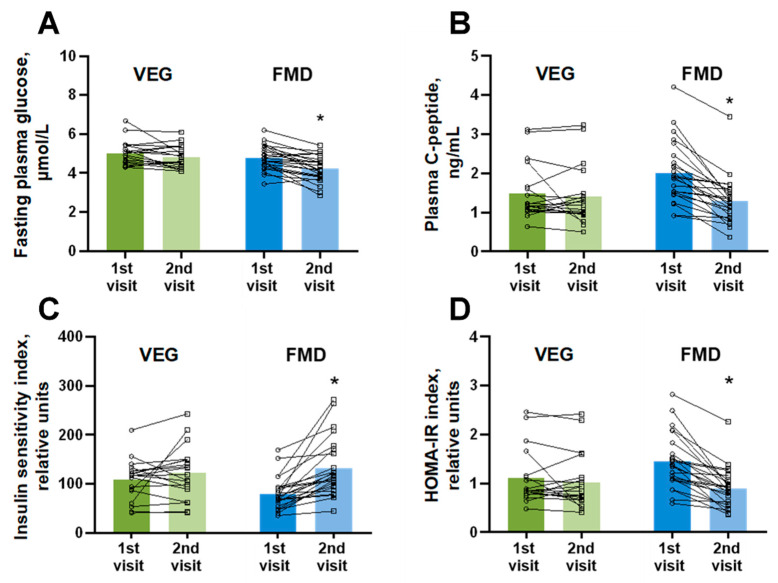
Changes in fasting plasma glucose levels (**A**), plasma C-peptide levels (**B**), insulin sensitivity index (**C**), and HOMA-IR index (**D**) after the 5-day cycle of regular diet supplemented with additional vegetables (VEG) and fasting-mimicking diet (FMD). The results are presented as the mean and independent values of 19 volunteers in the VEG group and 24 volunteers in the FMD group. * Indicates a significant difference from the respective group at the 1st visit (Wilcoxon matched-pairs test), *p* < 0.05.

Next, we evaluated the effects of a 5-day cycle of the VEG diet and FMD on metabolic parameters. Fasting plasma glucose (Figure 5A) in the VEG group was reduced by 0.22 ± 0.12 mmol/L. Meanwhile, in the FMD group, the lowering of fasting plasma glucose was 2.7 times more pronounced (a decrease of 0.57 ± 0.11 mmol/L). A similar pattern was observed in plasma C-peptide levels (Figure 5B), where the FMD group exhibited a significant reduction in plasma C-peptide compared to the VEG group (a decrease of 0.72 ± 0.11 ng/mL and 0.09 ± 0.11 ng/mL, respectively).

Subsequently, volunteers in the FMD group also had an improved insulin sensitivity index (Figure 5C). The increase in insulin sensitivity was 3.8 times greater than that in the VEG group and exceeded the baseline measurement by more than 60%. The benefits of FMD were even more pronounced when we calculated the HOMA-IR index, which defines the extent of insulin resistance (Figure 5D). In the VEG group, we observed a nonsignificant reduction in HOMA-IR by 0.08 ± 0.08 units. In contrast, every volunteer in the FMD group showed a reduced HOMA-IR index, with an average decrease of 0.55 ± 0.08 units.

In the FMD group, we also observed a slight reduction in plasma high-density lipoprotein (HDL) levels after the 5-day dietary intervention. Both diets showed similar effects on plasma triglycerides (a reduction of up to 15%). However, no other significant changes in the plasma lipid profile were evident in any of the experimental groups (Table 2).

## 4. Discussion

In the present study, we demonstrate that 5 days of FMD is a viable dietary strategy to reduce plasma levels of TMAO, which is a diet-derived cardiovascular and metabolic disease risk biomarker. Moreover, our data suggest that the reduction in TMAO and improvement in the parameters that characterize glucose metabolism and the general metabolic state in healthy volunteers are attributed to intermittent energy restriction and the limitation of animal-derived protein consumption rather than increased vegetable intake.

The baseline characteristics of the volunteers in our study showed that they were slightly overweight and had plasma levels of TMAO that ranged from low values to extremely high values that are way above the CVD risk threshold (up to 24 μmol/L), indicating the high individual variability of TMAO [40]. Because of this, fasting plasma glucose, as one of the main parameters characterizing metabolic health, was chosen as the key criterion for randomization in our study. Nevertheless, the adherence to FMD resulted in a significant decrease in plasma TMAO levels in 75% of the volunteers. At the endpoint, 22 out of 24 volunteers had plasma TMAO levels below the CVD risk threshold in the FMD group. Moreover, a recent study reported that the benefits of FMD are more pronounced in individuals at risk than in those whose metabolic markers are within the normal range [41], which is in line with our findings. The same applied to TMAO levels, as the most noticeable reduction in TMAO concentrations was also observed in volunteers of the FMD group with higher baseline TMAO plasma concentrations.

In addition to the beneficial effects attributed to FMD, such as a decrease in fasting plasma glucose, reduction in C-peptide concentrations and overall improvement of metabolic health, the volunteers in the FMD group also presented a slight reduction in HDL levels, which may raise concerns about the development of atherosclerosis [42]. However, FMD can be defined as a very low-calorie diet (VLCD), as the caloric intake is ~800 kCal [43] for 4 subsequent days. Although previously reported studies applying VLCD are very heterogeneous, there is some evidence that adherence to VLCD can result in a decrease in HDL levels; however, after the completion of VLCD, HDL levels tend to regain previous levels or even surpass them [44], which was also evident in the pilot data of our study (data not shown). Therefore, based on the cyclic and short-term regimen of FMD, we believe it should not be considered detrimental in terms of the reduction in HDL levels and development of atherosclerosis.

Although the data from observational studies suggest that increased vegetable intake is also inversely associated with biomarkers of metabolic diseases [45,46], these findings are poorly supported by the evidence from interventional studies [47]. Our results also indicate that a short-term increase in vegetable intake, as in the VEG group, may not be sufficient to reduce plasma TMAO levels and provide noticeable benefits with respect to metabolic health, as we only observed a significant reduction in plasma triglyceride levels in the VEG group. Moreover, volunteers in the VEG group were expected to proceed with their usual caloric intake and dietary habits in terms of meat consumption, which has been associated with an increased risk of metabolic syndrome [48,49] and T2D [50]. An alternative to FMD, in terms of limiting the consumption of products of animal origin, would be a vegan diet, which in a recent study displayed promising results and reduced plasma TMAO levels already a week after switching to a plant-based diet [51]. However, the TMAO concentration returned to the previous level after the reintroduction of the usual diet [51], indicating that a vegan diet should be used as a permanent dietary regimen to sustain TMAO levels within the normal range. This in turn could lead to lowered compliance with the diet [52], a problem previously reported with continuous caloric restriction as well [23,24,25]. FMD, on the other hand, due to its cyclic nature, is associated with high compliance [53], which we also observed in our study. Overall, our previous and present observations emphasize the importance of reduced animal-derived protein consumption and limited calorie intake to achieve beneficial results, as in the case of FMD.

The main limitation of our study is the short-term nature of the designated dietary interventions (for only a 5-day period) imitating an acute change in diet. However, it has already been reported that such 5-day cycles of FMD could also serve as a long-term strategy if repeated each month [28]. Since our pilot study indicates that, to some extent, the reduction in TMAO levels in plasma can also be observed a week after the completion of the FMD cycle (data not shown), further research should be conducted to assess the durability of the beneficial effects of FMD on TMAO levels in the plasma after returning to the usual diet. As the production of TMA is strictly microbiota-dependent, another limitation is that we were not able to collect samples to assess the impact of FMD on gut microbiota. Some studies state that alterations in microbiota composition that favour TMA-producing bacteria are a possible mechanism by which plasma TMAO levels increase in T2D patients [54,55]. However, recent research shows that some of the typical deviations observed in gut microbiota composition in patients with T2D [56,57] or atherosclerosis [58] can be restored by FMD [59,60], thus possibly lowering TMA production and reducing CVD risks. Overall, these data suggest that the benefits of FMD are not limited to only the exclusion of dietary sources of TMAO [61,62,63], but could also be explained through the impact on gut microbiota composition. Moreover, it would also be of great interest to investigate the changes in the abundance of specific TMA-producing bacterial genera after following the FMD cycle and upon reintroduction of the usual diet, as it has been shown that some of the beneficial effects on gut microbiota composition occur only after continuation of the usual diet [60].

To conclude, our results show that FMD, a vegetable-based, low-calorie variation of intermittent fasting with a strict exclusion of animal-derived protein sources, is an efficient strategy to reduce plasma TMAO levels. Our results add a novel component to the interaction of FMD and the metabolic state of a person, suggesting that TMAO reduction should be considered one of the noteworthy benefits of FMD with respect to improving metabolic health. However, further research is needed to assess the potential of compliance to FMD and the effects on TMAO levels after the completion of several cycles of the diet, as well as upon the reintroduction of the regular diet.

## Figures and Tables

**Table 1:** Baseline data characterizing bio-anthropometric and biochemical parameters of the study participants.

Baseline Characteristics	VEG (*n* = 19)	FMD (*n* = 24)	*p*-Value
Age, years	37 ± 3	39 ± 2	0.660
Sex, *n* (%)			
Men	6 (31.6)	9 (37.5)	
Women	13 (68.4)	15 (62.5)	
BMI, kg/m^2^	25.2 ± 0.9	28.8 ± 0.9	0.004
Body type (regional fat distribution), *n* (%)			
Abdominal	8 (42.1)	10 (41.7)	
Gluteofemoral	11 (57.9)	14 (58.3)	
Plasma biochemistry			
Hemoglobin, g/L	144.0 ± 3.5	150.3 ± 6.2	0.350
Glucose, mmol/L	4.99 ± 0.13	4.87 ± 0.11	0.470
HDL cholesterol, mmol/L	1.51 ± 0.07	1.49 ± 0.08	0.841
LDL cholesterol, mmol/L	3.33 ± 0.16	3.37 ± 0.19	0.857
Triglycerides, mmol/L	1.44 ± 0.22	1.30 ± 0.09	0.440
Creatinine, μmol/L	75.7 ± 3.8	75.2 ± 6.6	0.941
eGFR, mL/min/1.73 m^2^	86.6 ± 5.9	92.7 ± 6.9	0.527
ALT, U/L	21.5 ± 2.7	24.7 ± 3.4	0.478
Total bilirubin, μmol/L	9.7 ± 1.1	10.3 ± 1.5	0.788
Lipase, U/L	41.6 ± 1.9	37.3 ± 1.8	0.149
ESR, mm/h	2.9 ± 0.9	2.3 ± 0.2	0.641
CRP, mg/L	1.26 ± 0.33	1.24 ± 0.22	0.964
TMAO, μmol/L	3.65 ± 0.68	6.22 ± 1.16	0.083
Physical activity, *n* (%)			
Low	11 (57.9)	15 (62.5)	
Moderate	6 (31.6)	8 (33.3)	
High	2 (10.5)	1 (4.2)	
Meat consumption, *n* (%)			
>5 servings per week	10 (52.6)	14 (58.3)	
3–5 servings per week	9 (47.4)	9 (37.5)	
<3 servings per week	0 (0.0)	1 (4.2)	

Data are presented as the mean ± SEM, unless indicated otherwise. ALT, Alanine aminotransferase/Glutamate pyruvate transaminase; BMI, body mass index; CRP, C-reactive protein; eGFR, estimated glomerular filtration rate; ESR, Erythrocyte Sedimentation Rate; HDL, high-density lipoprotein; LDL, low-density lipoprotein; TMAO, trimethylamine N-oxide.

**Table 2:** The effects of a 5-day regular diet supplemented with 4 servings of vegetables (VEG) and fasting-mimicking diet (FMD) on the plasma lipid profile.

	VEG		FMD	
	1st Visit	2nd Visit	1st Visit	2nd Visit
High-density lipoprotein, μmol/L	1.51 ± 0.07	1.51 ± 0.07	1.49 ± 0.08	1.30 ± 0.07
Low-density lipoprotein, μmol/L	3.33 ± 0.16	3.32 ± 0.15	3.38 ± 0.19	3.41 ± 0.20
Triglycerides, μmol/L	1.44 ± 0.22	1.22 ± 0.20 *	1.30 ± 0.09	1.10 ± 0.07 *

The results are presented as the mean ± SEM of 19 volunteers in the VEG group and 24 volunteers in the FMD group. * Indicates a significant difference from the respective group at the 1st visit (Wilcoxon matched-pairs test), *p* < 0.05.

## Data Availability

The datasets generated and analyzed during the current study are available from the corresponding author on reasonable request.

## Supplementary Materials

The following are available online at https://www.mdpi.com/article/10.3390/nu14051093/s1, Table S1: The original data sample of TMAO detection using UPLC/MS/MS analysis.
