# Supplementary material for: Fasting-Mimicking Diet Reduces Trimethylamine N-Oxide Levels and Improves Serum Biochemical Parameters in Healthy Volunteers
Source: Nutrients. 2022 Mar 5;14(5):1093. doi: 10.3390/nu14051093 (PMC8912301; doi:10.3390/nu14051093)
Supplement: Supplementary file 1 [file nutrients-14-01093-s001.zip › nutrients-1556920-supplementary.pdf]

Supplementary Table 1. The original data sample of TMAO detection using UPLC/MS/MS analysis.

	Sample Text	Type	Std. Conc	RT	Area	Height	IS Area	Response	Detection Flags	Conc.	%Dev
1	Std0			1.71	66.484	1024	42452	0.00157	bb	0.02	
2	Std1	Standard	0.17	1.71	324.428	5056	42631	0.00761	bb	0.19	11
3	Std1	Standard	0.17	1.71	318.034	4892	43164	0.00737	bb	0.18	7.1
4	Std1	Standard	0.17	1.71	313.148	4946	42850	0.00731	bb	0.18	6.1
5	Std2	Standard	0.34	1.71	568.378	8869	43120	0.01318	bb	0.34	0.7
6	Std2	Standard	0.34	1.71	558.466	8666	43534	0.01283	bb	0.33	-2.2
7	Std2	Standard	0.34	1.71	535.685	8276	43089	0.01243	bb	0.32	-5.4
8	Std3	Standard	0.68	1.71	1046.538	16159	42155	0.02483	bb	0.66	-2.5
9	Std3	Standard	0.68	1.71	998.805	15447	41416	0.02412	bb	0.64	-5.4
10	Std3	Standard	0.68	1.71	1041.152	16218	42047	0.02476	bb	0.66	-2.7
11	Std4	Standard	3.25	1.71	4931.394	77089	41633	0.11845	bb	3.23	-0.8
12	Std4	Standard	3.25	1.71	4813.635	75055	42246	0.11394	bb	3.1	-4.5
13	Std4	Standard	3.25	1.71	4863.459	75288	42179	0.1153	bb	3.14	-3.4
14	Std5	Standard	13.67	1.71	20547.32	318532	39672	0.51793	bb	13.85	1.3
15	Std5	Standard	13.67	1.71	19921.92	308757	39590	0.50321	bb	13.47	-1.5
16	Std5	Standard	13.67	1.71	20217.15	315503	39122	0.51678	bb	13.82	1.1
17	Std6	Standard	34.18	1.71	48451.29	743715	36122	1.34133	bb	34.4	0.6
18	Std6	Standard	34.18	1.71	47198.57	721789	35699	1.32213	bb	33.94	-0.7
19	Std6	Standard	34.18	1.71	47897.53	736975	35520	1.34847	bb	34.57	1.1
20	Std7	Standard	68.35	1.71	86924.29	1315792	30450	2.85468	bb	68.46	0.2
21	Std7	Standard	68.35	1.71	87341.48	1318469	30275	2.88497	bb	69.1	1.1
22	Std7	Standard	68.35	1.71	85197.59	1288035	30420	2.80071	bb	67.31	-1.5
23	Std0			1.71	83.691	1282	41345	0.00202	bb	0.03	
24	Blank			1.71	71.725	1089			bb		
25	QC Std3	QC	0.68	1.71	1009.712	15651	41067	0.02459	bb	0.66	-3.5
26	QC Std5	QC	13.67	1.71	20290.67	315454	39380	0.51526	bb	13.78	0.8
27	Blank			1.71	79.463	1210			bb		
28	Lo-01			1.71	6039.451	93347	44396	0.13604	bb	3.7	
29	Lo-01			1.71	6133.015	94523	45674	0.13428	bb	3.66	

30	Lo-02			1.71	3505.479	53543	44729	0.07837	bb	2.13	
31	Lo-02			1.71	3537.478	54899	45730	0.07736	bb	2.1	
32	Lo-03			1.71	4686.411	72447	45533	0.10292	bb	2.8	
33	Lo-03			1.71	4785.255	73613	45710	0.10469	bb	2.85	
34	Lo-04			1.71	2955.973	45281	46542	0.06351	bb	1.73	
35	Lo-04			1.71	2885.156	44306	45989	0.06274	bb	1.7	
36	Lo-05			1.71	4006.632	62056	45709	0.08765	bb	2.39	
37	Lo-05			1.71	4036.774	62502	46961	0.08596	bb	2.34	
38	Lo-06			1.71	19850.93	305676	44122	0.44991	bb	12.08	
39	Lo-06			1.71	20135.51	311505	44134	0.45623	bb	12.24	
40	Lo-07			1.71	4166.833	63923	47797	0.08718	bb	2.37	
41	Lo-07			1.71	4121.874	63660	46894	0.0879	bb	2.39	
42	Lo-08			1.71	2289.944	35184	46832	0.0489	bb	1.32	
43	Lo-08			1.71	2243.637	34534	46825	0.04792	bb	1.3	
44	Lo-09			1.71	5357.708	83189	46809	0.11446	bb	3.12	
45	Lo-09			1.71	5317.834	81884	46459	0.11446	bb	3.12	
46	Lo-10			1.71	8597.462	132868	45590	0.18858	bb	5.13	
47	Lo-10			1.71	8833.816	137076	47388	0.18641	bb	5.07	
48	Lo-11			1.71	3383.108	52558	48471	0.0698	bb	1.9	
49	Lo-11			1.71	3413.598	52504	49421	0.06907	bb	1.88	
50	Lo-12			1.71	5792.995	88733	47078	0.12305	bb	3.35	
51	Lo-12			1.71	5834.93	89771	48009	0.12154	bb	3.31	
52	Blank			1.71	82.627	1226			bb		
53	QC Std3	QC	0.68	1.71	1155.614	17928	46965	0.02461	bb	0.66	-3.4
54	QC Std5	QC	13.67	1.71	21911.34	340872	43667	0.50179	bb	13.43	-1.7
55	Blank			1.71	80.74	1246			bb		
56	Lo-01-2			1.71	4168.061	64315	47147	0.08841	bb	2.41	
57	Lo-01-2			1.71	4207.027	65355	48003	0.08764	bb	2.39	
58	Lo-02-2			1.71	2986.733	46315	48982	0.06098	bb	1.66	
59	Lo-02-2			1.71	2917.715	44912	49666	0.05875	bb	1.59	
60	Lo-03-2			1.71	5451.363	83660	48030	0.1135	bb	3.09	
61	Lo-03-2			1.71	5518.889	84765	47226	0.11686	bb	3.18	
62	Lo-04-2			1.71	6431.987	98688	46938	0.13703	bb	3.73	

63	Lo-04-2			1.71	6450.63	99273	47897	0.13468	bb	3.67	
64	Lo-05-2			1.71	2888.207	44637	48649	0.05937	bb	1.61	
65	Lo-05-2			1.71	2917.39	44821	47915	0.06089	bb	1.65	
66	Lo-06-2			1.71	3372.784	51417	48651	0.06933	bb	1.88	
67	Lo-06-2			1.71	3541.581	54467	47897	0.07394	bb	2.01	
68	Lo-07-2			1.71	20496.96	313640	44936	0.45613	bb	12.24	
69	Lo-07-2			1.71	20825.82	318982	44103	0.4722	bb	12.66	
70	Lo-08-2			1.71	3405.297	52159	49493	0.0688	bb	1.87	
71	Lo-08-2			1.71	3410.956	52474	49949	0.06829	bb	1.86	
72	Lo-09-2			1.71	5120.767	78983	49190	0.1041	bb	2.83	
73	Lo-09-2			1.71	5146.862	79199	49095	0.10484	bb	2.85	
74	Lo-10-2			1.71	8035.383	123480	47548	0.16899	bb	4.6	
75	Lo-10-2			1.71	8086.355	124550	48093	0.16814	bb	4.57	
76	Lo-11-2			1.71	5104.56	78044	49863	0.10237	bb	2.79	
77	Lo-11-2			1.71	5260.009	81421	49741	0.10575	bb	2.88	
78	Lo-12-2			1.71	3190.514	49229	49881	0.06396	bb	1.74	
79	Lo-12-2			1.71	3178.883	48411	48758	0.0652	bb	1.77	
80	Blank			1.71	85.725	1340			bb		
81	QC Std3	QC	0.68	1.71	1146.247	17778	47589	0.02409	bb	0.64	-5.5
82	QC Std5	QC	13.67	1.71	22408.66	348464	44848	0.49966	bb	13.38	-2.1
83	Blank			1.71	82.903	1238			bb		

Quantification was carried out using the ratio of the peak areas of the sample and the internal standard (IS), based on the standard curve prepared using trimethylamine N-oxide (TMAO) (Alfa Aeser, Ward Hill, Massachusetts, USA) in a range of concentrations up to 68.35 μmol/L. All standard curve concentrations are assayed in triplicate; test samples are assayed in duplicate. Between different sets of samples, additional quality control (QC) and blank samples are assayed.
